# In–out versus out–in technique for ACL reconstruction: a prospective clinical and radiological comparison

**DOI:** 10.1007/s10195-017-0458-7

**Published:** 2017-05-08

**Authors:** Edoardo Monaco, Mattia Fabbri, Andrea Redler, Raffaele Iorio, Jacopo Conteduca, Giuseppe Argento, Andrea Ferretti

**Affiliations:** Orthopaedic Department and “Kirk Kilgour” Sports Injury Center, Sant’ Andrea Hospital, “La Sapienza”, University of Rome, Via di Grottarossa, 1035-1039 Rome, Italy

**Keywords:** ACL, Femoral tunnel, Transtibial, Out–in technique

## Abstract

**Background:**

Several studies have recently shown better restoration of normal knee kinematics and improvement of rotator knee stability after reconstruction with higher femoral tunnel obliquity. The aim of this study is to evaluate tunnel obliquity, length, and posterior wall blowout in single-bundle anterior cruciate ligament (ACL) reconstruction, comparing the transtibial (TT) technique and the out–in (OI) technique.

**Materials and methods:**

Forty consecutive patients operated on for ACL reconstruction with hamstrings were randomly divided into two groups: group A underwent a TT technique, while group B underwent an OI technique. At mean follow-up of 10 months, clinical results and obliquity, length, and posterior wall blowout of femoral tunnels in sagittal and coronal planes using computed tomography (CT) scan were assessed.

**Results:**

In sagittal plane, femoral tunnel obliquity was 38.6 ± 10.2° in group A and 36.6 ± 11.8° in group B (*p* = 0.63). In coronal plane, femoral tunnel obliquity was 57.8 ± 5.8° in group A and 35.8 ± 8.2° in group B (*p* = 0.009). Mean tunnel length was 40.3 ± 1.2 mm in group A and 32.9 ± 2.3 mm in group B (*p* = 0.01). No cases of posterior wall compromise were observed in any patient of either group. Clinical results were not significantly different between the two groups.

**Conclusions:**

The OI technique provides greater obliquity of the femoral tunnel in coronal plane, along with satisfactory length of the tunnel and lack of posterior wall compromise.

**Level of evidence:**

II, prospective study.

## Introduction

Although many studies have reported good results in the short term after anterior cruciate ligament (ACL) reconstruction, some concerns still remain. Historically, long-term studies, not including recent knowledge on anatomical femoral tunnel placement through the transportal (TP) technique, have reported high incidence of joint degeneration (as much as 52–56 % at 12–13 years after surgery) [1, 2], and an estimated 8–10 % of reconstructions result in recurrent instability and in graft failure. Several authors identify improper femoral tunnel placement as a common reason of failure. The anatomical insertion of the ACL on the femur lies very low in the notch, spreading between 11 and 9–8 o’clock, and the center lies lower than 11 o’clock position [3]. Recommendations for femoral tunnel placement include the over-the-top position [4], the central part [5], and the posterosuperior part of the insertion area [6]. Frequently, grafts are placed too far anterior on the femur, resulting in a vertically oriented graft [7]. Correct position of the femoral tunnel has a great influence on knee kinematics and is considered a key factor for successful single-bundle ACL reconstruction. Correct position in sagittal plane of the graft in ACL reconstruction has been recognized as critical for restoration of normal knee kinematics [8]. A 62.5 % incidence of graft failure can be expected when the femoral tunnel is placed anterior [9]. However, the importance of correct position of an ACL graft in coronal plane has been underestimated. In recent years, many authors have demonstrated biomechanical advantages of recreating the obliquity of the ACL graft in coronal plane [7, 10, 11]. Furthermore, it has been shown that a vertically oriented graft in coronal plane is associated with poor clinical results, resulting in persistent pivot shift [12]. Moreover, in recent years, many authors have shown better restoration of normal knee kinematics and improvement of rotator knee stability after reconstruction with higher femoral tunnel obliquity [10, 11]. An oblique femoral tunnel controls anterior tibial translation and internal tibial rotation, which may correlate clinically with an absent pivot shift.

With the introduction of arthroscopic-assisted ACL reconstructions, different techniques for femoral tunnel creation have been developed. The most popular technique for femoral tunnel creation in ACL reconstruction is the transtibial (TT) technique [13]. This technique has the advantage of effecting an isometric, or near-isometric, graft throughout knee range of motion [14]. However, advances in anatomy and biomechanics of the knee have shifted the concept of proper femoral tunnel position from the isometric point to restoration of the anatomy of the ACL. It is well documented that the ACL is made of two different bundles, the anteromedial (AM) and posterolateral (PL), with different specific functions, as the AM bundle controls anteroposterior laxity whereas the PL bundle ensures rotational stability, but working synergically so that they cannot be considered as separate structures [15, 16]. Therefore, double-bundle reconstructions have been proposed to replicate the anatomy of the native ACL, with literature showing no definitive clinical superiority over single-bundle techniques [17, 18]. Recent studies have discussed the inability of TT drilling technique to accurately position femoral tunnels within native ACL insertion sites [3, 19–22] due to an inability to freely position the femoral tunnel, as it is predetermined by the tibial tunnel placement, allowing for limited adjustment [14, 23]. Independent drilling techniques, such as TP and out–in (OI) techniques, have been developed to achieve more accurate femoral tunnels independently from the tibial tunnels. With these techniques, the orientation of the femoral tunnel becomes more oblique in the coronal plane than with the TT technique, with the potential advantage of preventing anterior translation and internal rotation of the tibia, as suggested by some recent biomechanical studies [24–27].

The TP technique allows the femoral tunnel to be reamed through the anteromedial portal or, as suggested by some authors [28], creation of an accessory anteromedial portal as inferior (close to the tibia) as possible for viewing the femoral footprint [13, 29]. In this way, the surgeon has more freedom to place the graft in the anatomical position at 10 o’clock.

The double incision is the oldest and perhaps easiest technique, but a second lateral incision is required.

The goal of this prospective study is to evaluate tunnel obliquity, length, and posterior wall compromise in single-bundle ACL reconstruction, comparing the TT in–out technique and the two-incision OI technique. Our hypothesis is that the OI technique provides more oblique placement of the graft, closer to the anatomy of the ACL, in comparison with the TT technique.

## Materials and methods

Patients admitted from September 2014 to April 2015 with diagnosis of ACL tear were enrolled in this study. All patients were carefully evaluated; clinical assessment included Lachman, pivot shift, and varus/valgus tests as well as investigation of meniscal tears. Forty consecutive patients (26 male, 14 female) gave consent for inclusion in this study. Inclusion criteria were: chronic ACL tear (>2 months from injury); ACL tear revealed by positive Lachman and pivot shift test (+ to +++). Exclusion criteria were: multiligamentous associated injuries as detected by clinical examination (varus or valgus stress and posterior drawer test positive) or magnetic resonance imaging (MRI); previous knee surgery; age >40 years; body mass index (BMI) >30 kg/m^2^. Patients with meniscal tear or cartilage damage were included in the study.

The forty patients were randomly divided into two groups: in group A (20 patients), reconstruction was performed with a standard TT in–out technique. The tibial tunnel was always drilled using a guide wire at 65° on the sagittal plane and 30° on the axial plane. Graft fixation was performed with a bioabsorbable screw (BioRCI-HA) on the tibial side and with Endobutton (Smith and Nephew) on the femoral side.

In group B (20 patients), a two-incision OI technique was performed: the tibial tunnel was drilled using a guide wire, and the tibial guide was adjusted at 65° on the sagittal plane and 30° on the axial plane. The femoral tunnel was drilled through a second lateral small incision in an out–in manner. Tibial fixation was performed with Evolgate (Citieffe, Bologna, Italy) and femoral fixation with Swing-Bridge (Citieffe, Bologna, Italy). In both groups, a doubled gracilis–semitendinosus tendon (DGST) graft was used and all procedures were performed by the same surgeon (A.F.).

All patients underwent standardized evaluation at follow-up. This included assessment on Tegner and Lysholm scales, International Knee Documentation Committee (IKDC), and KT-1000 (MEDmetric, San Diego, CA) knee arthrometric evaluation.

**Fig. 1:**
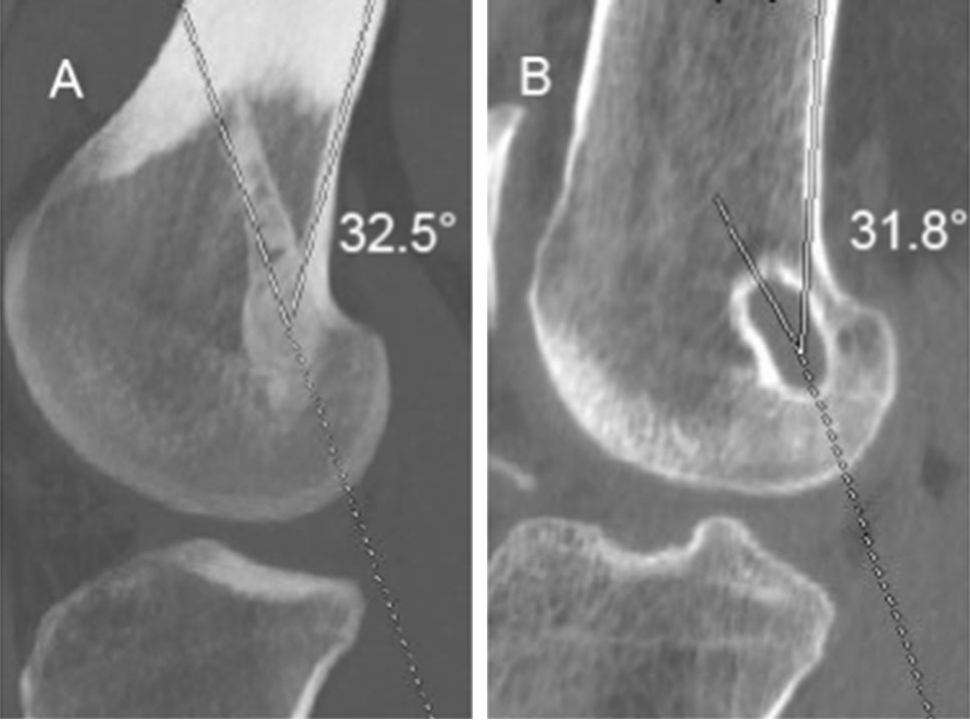
Sagittal obliquity defined by the angle subtended between the tunnel and longitudinal axis of the femur. In this case, the angle is 32.5° for the in–out group (**a**) and 31.8° for the out–in group (**b**)

**Fig. 2:**
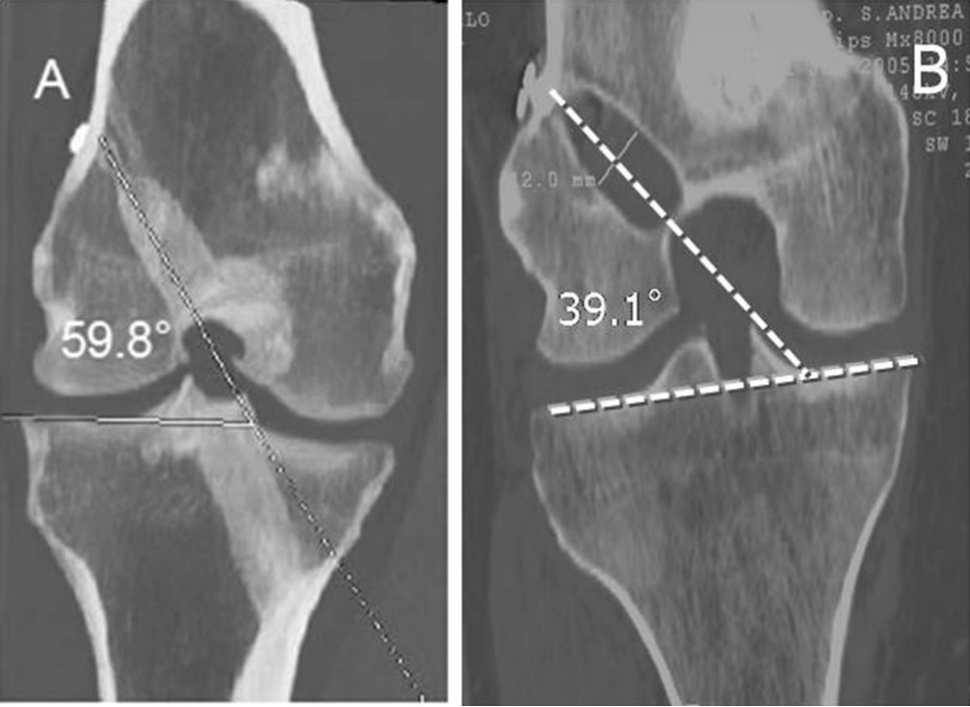
The coronal obliquity is shown on these CT images. A line parallel to the joint line and the femoral tunnel was used to calculate the coronal obliquity. In this case the value is 59.8° for the in–out group (**a**) and 39.1° for the out–in group (**b**)

Moreover, CT evaluation was performed with a 16-slice Philips MX 8000 MSCT scanner with postprocessing multislab reconstruction on sagittal and coronal plane. MSCT scanning was carried out from a level just above the femoral external foramen to a level below the outer hole of the tibial tunnel in order to visualize the position of the fixation device. The slice thickness was 1 mm, with retroreconstruction of 0.75 mm made in all patients before postprocessing imaging with multislab views. The obliquity, length, and posterior wall blowout of femoral tunnels were assessed. In sagittal plane, obliquity was defined by the angle subtended between the tunnel and longitudinal axis of the femur (Fig. 1). In coronal plane, the obliquity was defined by the angle subtended between the femoral tunnel and the joint line (Fig. 2).

**Fig. 3:**
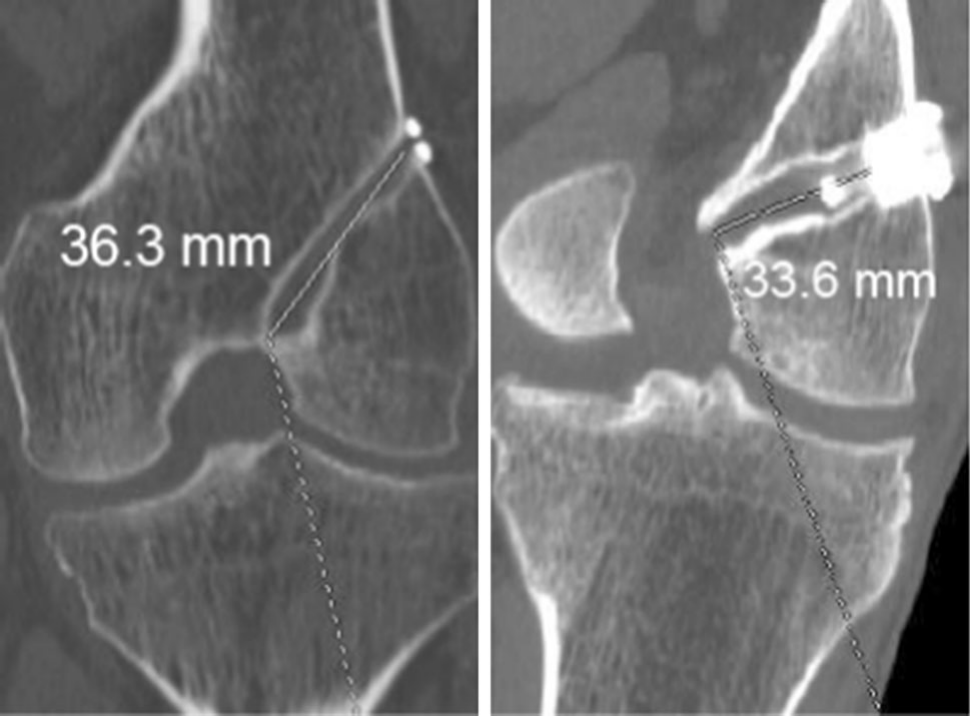
Tunnel length was evaluated on selected images, calculating from intra-articular to extra-articular aperture

Tunnel length was evaluated on selected images, calculating from intra-articular to extra-articular aperture (Fig. 3). Posterior wall blowout was defined by any breach in the posterior cortical wall of the tunnel. A senior musculoskeletal radiologist and an orthopedic surgeon performed all the measurements.

The data obtained from the study were analyzed using the chi-squared test and Fisher exact test. *p*-Value less than 0.05 was considered significant.

## Results

No differences were found between the baseline characteristics of the two groups (Table 1). No postoperative complications were recorded in either group.

**Table 1:** Baseline characteristics

Variable	Group A	Group B
Age (years)*	32.5 ± 4.7	31.7 ± 5.1
Sex (M; F)	15; 5	14; 6
Dominant side involvement	13	13
Follow-up (months)*	10.1 ± 1.7	10.8 ± 1.5
Meniscal lesions (medial; lateral)	2; 3	2; 4
Chondral lesions (femur; tibia)	2; 0	1; 0

* Data presented as mean ± standard deviation

In group A (20 patients), mean Lysholm score was 55.4 ± 9.4 preoperatively and 96.2 ± 3.3 points at follow-up, with a decrease in mean Tegner value from 7.5 (range 5–10) before surgery to 6.5 (range 4–10) at follow-up. Mean IKDC form at follow-up was 94.9 ± 3.8. Specifically, according to IKDC Knee Examination Form, 16 patients (80 %) were detected in group A (normal) and 4 patients (20 %) were detected in group B (nearly normal). Lachman test was evaluated as negative in all patients (100 %); Pivot-shift test was found to be negative (grade 0) in 16 patients (80 %) and positive (grade 1) in 4 patients (20 %). Arthrometric evaluation showed mean side-to-side difference of 1.8 mm (range 0.3–2.3 mm) at maximum manual handling between the involved and contralateral healthy knee. Sixteen out of 20 patients (80 %) returned to preinjury level of sport activities at a mean of 8.4 ± 1.3 months postoperatively. The remaining four patients (20 %) had restricted their sport activities for reasons other than knee problems.

In group B (20 patients), the mean Lysholm score was 55.7 ± 19.2 preoperatively and 97.1 ± 2.8 points at follow-up, with a decrease in mean Tegner value from 7.2 (range 5–9) before surgery to 6.5 (range 4–9) at follow-up. Mean IKDC form at follow-up was 95.1 ± 5.3. Specifically, according to IKDC Knee Examination Form, 16 patients (80 %) were detected in group A (normal) and 4 patients (20 %) were detected in group B (nearly normal). Lachman test was evaluated as negative in all patients (100 %); Pivot-shift test was found to be negative (grade 0) in 18 patients (90 %) and positive (grade 1) in 2 patients (10 %). Arthrometric evaluation showed mean side-to-side difference of 1.7 mm (range 0.5–2 mm) at maximum manual handling between the involved and contralateral healthy knee. Seventeen out of 20 patients (85 %) had returned to preinjury level of sport activities at a mean of 8.1 ± 1.9 months postoperatively. The remaining three patients (15 %) had restricted their sport activities for reasons other than knee problems.

No significant differences were detected for any of the clinical parameters assessed between the two groups at follow-up (Table 2).

**Table 2:** Clinical findings at follow-up, comparison between groups

Variable	Group A	Group B	*p*-Value
Lysholm score*	96.2 ± 3.3	97.1 ± 2.8	0.46
Tegner*	6.5 ± 2.5	6.5 ± 2.5	0.36
IKDC*	94.9 ± 3.8	95.1 ± 5.3	0.08
Lachman	Negative (100 %)	Negative (100 %)	–
Pivot shift	Grade 0: 16/20 (80 %) Grade 1: 4/20 (20 %)	Grade 0: 18/20 (90 %) Grade 1: 2/20 10 %)	–
KT-1000 (mm)*	1.8 ± 1.5	1.7 ± 1	0.2

* Data presented as mean ± standard deviation

The radiological evaluation of group A showed mean femoral tunnel obliquity of 38.6 ± 10.2° in sagittal plane (Fig. 1a) and 57.8 ± 5.8° in coronal plane (Fig. 2a), and mean tunnel length of 40.3 ± 1.2 mm (Fig. 3a).

In group B, the mean femoral tunnel obliquity registered was 36.6 ± 11.8° in sagittal plane (Fig. 1b) and 35.8 ± 8.2° in coronal plane (Fig. 2b), and mean tunnel length was 32.9 ± 2.3 mm (Fig. 3b).

Statistical analysis showed a significant difference between the two groups in femoral tunnel obliquity in coronal plane (*p* = 0.009), with a more oblique femoral tunnel placement registered in group B. Conversely, no significant differences were found when comparing femoral tunnel obliquity in sagittal plane between the two groups (*p* = 0.62).

Comparing mean femoral tunnel length between the two groups, the statistical analysis showed a significant difference (*p* = 0.01), with longer femoral tunnel registered in group A (Table 3).

**Table 3:** Radiological findings at follow-up, comparison between groups

Variable	Group A	Group B	*p* Value
Femoral tunnel obliquity in sagittal plane	38.6 ± 10.2°	36.6 ± 11.8°	0.62
Femoral tunnel obliquity in coronal plane	57.8 ± 5.8°	35.8 ± 8.2°	**0.009**
Femoral tunnel length	40.3 ± 1.2 mm	32.9 ± 2.3 mm	**0.01**

* Data presented as mean ± standard deviation

Bold values correspond to statistcal significance

In both groups, no cases of posterior wall blowout were observed.

## Discussion

The most important finding of this study is that a significant difference in femoral tunnel obliquity in the coronal plane was found when comparing the TT technique with the OI technique. Specifically, in the coronal plane, TT drilling resulted in a more vertical femoral tunnel placement while femoral tunnels drilled with an OI technique were found to be more oblique, with approximately 20° greater obliquity in coronal plane in comparison with TT drilling. Therefore, the hypothesis of the study was confirmed. Moreover, no cases of posterior wall blowout were observed, with no cases of tunnel length less than 25 mm, in both groups. However, this finding did not result in differences in the clinical outcomes between the two groups at mean follow-up of 10 months.

Correct selection of the femoral tunnel position is a critical step in ACL reconstruction. The effect of different placement of the femoral tunnel has been evaluated by many authors. A femoral tunnel placed at 11 o’clock in the intercondylar notch has been considered the standard and has been accepted as the correct tunnel location for all individuals [30, 31]. However, as the ACL does not function as a simple band of fibers with constant isometry, its structural complexity seems to be not completely restored by a reconstruction performed with this femoral placement. Moreover, 11 o’clock femoral placement seems to be insufficient to control complex rotatory loads [11]. According to biomechanical studies, oblique femoral tunnel placement in coronal plane results in better restoration of normal knee kinematics and improvement of rotator knee stability in comparison with a more vertical tunnel [10, 11] with no differences under combined rotary loads between double-bundle reconstruction and laterally placed single-bundle reconstruction [32].

Lee et al. [33], performing ACL reconstruction with a TT technique, reported that, in a subset of patients with vertical graft orientation, clinical examination (pivot shift, KT-1000 measurements) and Lysholm score were significantly worse in comparison with patients with a more oblique graft placement. Similarly, Jespen et al. [8] found that a change in the femoral tunnel placement, performed transtibially, from 1 o’clock position to 2 o’clock position (more oblique tunnel) resulted in a significant difference in the IKDC evaluation form. Furthermore, Carson et al. [34] suggested that a more vertical tunnel might not control internal tibial rotation, which could result in persistent instability. However, in our study, graft obliquity was not determined by the o’clock description because this system lacks precision and is highly dependent on subjective interpretation [35]. Moreover, none of the above-mentioned studies used an out–in technique to perform femoral tunnel drilling, so comparison with our results appears to be difficult.

The single-incision TT ACL reconstruction technique still seems to be the procedure most commonly performed by orthopedic surgeons [36]. Nevertheless, when using an in–out technique, the surgeon may not be able to place the tunnel within the margins of the anatomical ACL footprint [3]. In fact, femoral tunnel anatomical placement could be achieved if the starting point is close to the tibial joint line, resulting in a short tibial tunnel with concerns regarding sufficient tunnel length for graft fixation and graft incorporation [23]. Therefore, as suggested by some authors [37], if anatomical positioning of the femoral tunnel cannot be achieved with TT drilling, then an alternative approach may be indicated [38]. The TP technique has been shown to allow for slightly greater femoral tunnel obliquity compared with TT drilling [39]. However, a reported risk of the TP technique for ACL femoral tunnel creation is short tunnel length, which can result in reduced length of tendon graft within the femoral bone tunnel [40, 41]. This is an issue for surgeons desiring to avoid the risk of inadequate graft tissue within a tunnel, particularly when using suspensory fixation devices with fixed loop length, as the loop of the device leaves less length of graft within the tunnel. In fact, the drill angle in TP technique is somewhat constrained due to the combination of knee hyperflexion and portal fixed position just above the medial meniscus and lateral to the medial femoral condylar articular cartilage. On the other hand, the OI technique has the advantage of the flexibility of the over-the-top guides, which allow intraosseous distance measurement before drilling by observing marks on the guide pin sleeve. Therefore, if the distance is too short, manipulation of the drill angle and starting position can be performed to achieve a longer tunnel before drilling, whereas with the TP portal technique, intraosseous distance cannot be measured before pin passage.

The results of the present study show that OI femoral tunnel drilling achieved sufficient femoral tunnel length, with no cases of posterior wall blowout and no cases of tunnel length less than 25 mm. These results are in agreement with those previously reported, particularly by Lubowitz et al., who found, in a cadaveric model, mean OI tunnel length of 34.1 mm, with no cases of tunnel less than 25 mm long, when compared with TP technique [42].

An explanation of these results is that the two-incision OI technique allows the surgeon to center ACL footprint regardless of tibial tunnel placement or knee flexion angle. Thus, a more oblique femoral tunnel placement can be achieved, without the constraint of a guide through the tibial tunnel, limiting posterior tunnel blowout and shorter tunnel length, as knee flexion is not determinant for good visualization of the ACL femoral footprint.

This study has some limitations: First, the relatively small number of patients enrolled, which was in part due to the strict inclusion criteria; Second, the short-term follow-up, which does not allow clear determination of whether our results could affect clinical outcomes; Finally, it was not possible to correlate radiological and clinical results due to the relatively small number of patients enrolled.

However, while some authors [43] reported that bone tunnels are easily detectable on lateral radiographs, many others [44] reported problems concerning tunnel evaluation with X-rays, suggesting CT scan as a more accurate method to evaluate tunnel position [35].

Therefore, our use of CT scan for assessment of tunnel orientation with the advantages of a consecutive series of patients operated by the same surgeon using the same graft, as demonstrated by the small variation (narrow standard deviation) in tunnel positioning, could be considered a strength of the present study.

In conclusion, the results of the present study show that drilling the femoral tunnel with an OI technique results in greater obliquity of the femoral tunnel in coronal plane, as compared with the TT in–out technique. The OI technique seems to be a good option for single-bundle ACL reconstruction, when more oblique and anatomical femoral tunnel placement is desired to reduce the risk of short tunnel or posterior blowout.
